# AGGRESCAN and its evolution: A two-decade perspective on protein aggregation prediction

**DOI:** 10.1007/s12551-025-01404-9

**Published:** 2026-01-19

**Authors:** Giulia Pesce, Oriol Solé, Oriol Bárcenas, Salvador Ventura

**Affiliations:** 1 Institut de Biotecnologia i Biomedicina and Departament de Bioquímica i Biologia Molecular, Universitat Autònoma de Barcelona, Bellaterra, Barcelona 08193 Spain; 2 Institute of Advanced Chemistry of Catalonia (IQAC), CSIC, Barcelona, Spain; 3 Hospital Universitari Parc Taulí, Institut d’Investigació i Innovació Parc Taulí (I3PT-CERCA), Universitat Autònoma de Barcelona, Sabadell, Spain

**Keywords:** Protein aggregation, Aggregation-prone regions, Computational protein design, Amyloid, Molecular dynamics, Protein therapeutics

## Abstract

Protein aggregation is a widespread phenomenon with profound biological, biomedical, and biotechnological implications. In human disease, aberrant protein self-assembly is a hallmark of numerous neurodegenerative disorders, whereas in the biopharmaceutical industry, aggregation complicates the production, stability, and formulation of therapeutic proteins. The Aggrescan platform was one of the first empirically based tools designed to predict aggregation-prone regions (APRs) within protein sequences. It has since expanded to incorporate three-dimensional structural contexts and environmental conditions. This review provides a comprehensive overview of the development, application, and impact of the Aggrescan family of tools, which includes AGGRESCAN, Aggrescan3D, and the recent Aggrescan4D. We examine the algorithmic foundations, empirical validation, and key use cases spanning fields from biotechnology to biomedical research. Additionally, we describe how the recent integration of AlphaFold models has enabled proteome-scale exploration of aggregation determinants. This review highlights how Aggrescan has evolved alongside with advances in the field, becoming a reliable and accessible tool for studying and redesigning protein aggregation.

## Introduction

To perform its biological function, a protein must adopt a precise three-dimensional conformation. This native fold is encoded within the amino acid sequence and stabilized by a delicate balance of intramolecular interactions. Perturbations to this balance can trigger protein misfolding and aggregation, disrupting cellular proteostasis with far-reaching physiological consequences (Balchin et al. 2016). Both extrinsic and intrinsic factors influence aggregation behaviour. Environmental conditions—including pH shifts, ionic strength, redox state, and crowding—can modulate the solubility of proteins and expose latent aggregation determinants (McFarland et al. 2025; Thorlaksen et al. 2022; Zaman and Andreasen 2021; Zuo et al. 2021). Intrinsic factors such as point mutations, proteolytic clipping, alternative splicing, or post-translational modifications alter the local physicochemical microenvironment of the polypeptide chain, frequently unmasking or amplifying aggregation-prone regions (APRs) (Gadhavi et al. 2022; Li et al. 2024; Pandit et al. 2023; Pohl et al. 2020; Pounot et al. 2024). Importantly, in folded globular proteins, aggregation determinants are not restricted to sequentially contiguous stretches. Rather, spatially neighbouring residues that are distant in sequence may form aggregation-prone patches, underscoring the need for structurally informed prediction methods.

Protein aggregation is associated with neurodegenerative conditions such as Alzheimer’s and Parkinson’s diseases, where misfolded proteins accumulate into toxic oligomers, fibrils, and inclusions that compromise neuronal function and viability (Chinnathambi et al. 2025; Morris et al. 2024) In biotechnological and pharmaceutical settings, aggregation reduces the expression yield, stability, solubility, and developability of recombinant proteins, creating major bottlenecks in bioprocessing and formulation (Kannan et al. 2019; Wälchli et al. 2020). Thus, understanding and controlling aggregation is a unifying need across diverse scientific domains.

Given that the experimental characterization of aggregation is often demanding, costly, and system-specific, computational prediction has become a valuable complementary strategy for understanding this phenomenon. Early predictive algorithms, including TANGO, PASTA, WALTZ, and FoldAmyloid, established foundational principles by linking aggregation propensity to amino acid composition, β-structure formation, and physicochemical signatures (Fernandez-Escamilla et al. 2004; Flores-León et al. 2021; Garbuzynskiy et al. 2010; Louros et al. 2020; Walsh et al. 2014).

**Fig. 1:**
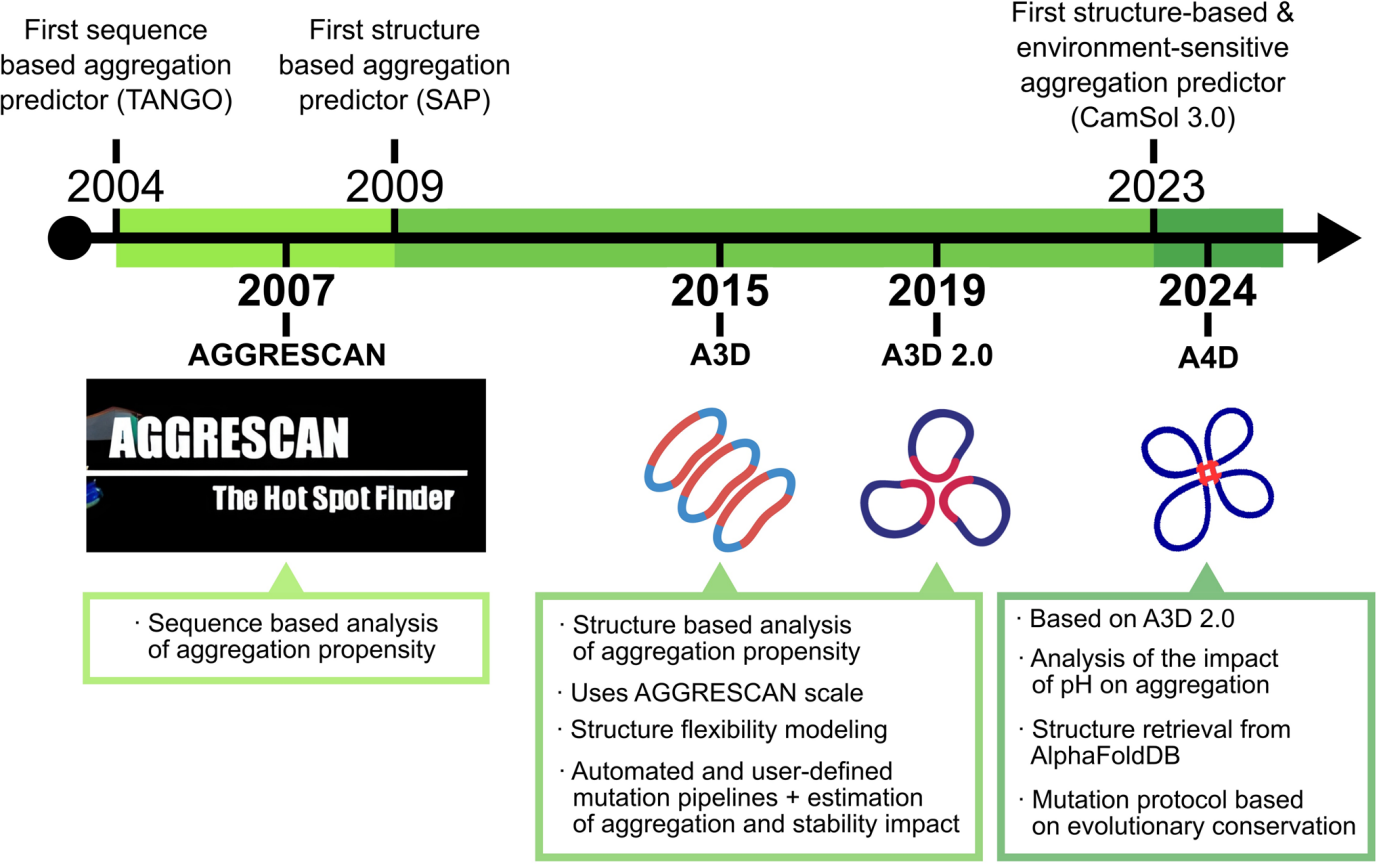
Timeline of Aggrescan releases in the context of protein aggregation predictors. The timeline begins with the release of TANGO in 2004 (Fernandez-Escamilla et al. 2004), closely followed by AGGRESCAN in 2007. In 2009, the field shifted from sequence-based predictors to predicting protein aggregation based on structure, trailblazed by SAP (Spatial aggregation propensity) (Chennamsetty et al. 2009). During this time, both Aggrescan3D (A3D) and Aggrescan3D 2.0 were released (2015 and 2019, respectively), expanding AGGRESCAN's capabilities to structured proteins, modeling their flexibility, and providing a comprehensive pipeline for virtual mutations and estimating their impact on aggregation and folding stability. Subsequently, CamSol3.0 (Oeller et al. 2023) sequence, released in 2023, initiated the field of structure-based, environment-sensitive aggregation prediction. A year later, Aggrescan4D (A4D) was released. A4D extends the A3D 2.0 algorithm by incorporating pH sensitivity, enabling structure retrieval directly from AlphaFoldDB, and introducing an additional mutation protocol informed by the evolutionary conservation of mutations

Introduced in 2007, AGGRESCAN is a sequence-based bioinformatic tool designed to identify aggregation-prone regions (APRs) using experimentally derived aggregation propensities obtained from in vivo studies (Fig. 1) (Conchillo-Solé et al. 2007; De Groot et al. 2012). This approach enabled an accurate and biologically relevant identification of APRs in both disease-associated and industrially significant proteins. Nevertheless, because AGGRESCAN relies on linear sequence information, its applicability to folded, globular proteins is limited, since potential aggregation sites can be structurally hidden or shielded in the native state, or formed by distant sequential patches that come together in the native structure.

To address these limitations, Aggrescan3D (A3D), first published in 2015 and later updated in 2019 as Aggrescan3D 2.0, expanded the original sequence-based framework to incorporate three-dimensional structural information (Fig. 1) (Zambrano et al. 2015; Kuriata et al. 2019b; Pujols et al. 2022; Badaczewska-Dawid et al. 2022). By projecting aggregation propensities onto protein structures, A3D enabled the identification of spatially adjacent solvent-exposed aggregation-prone patches (STAPs). It also introduced dynamic modelling capabilities to evaluate the effects of conformational flexibility on aggregation and a pipeline for mutational scanning. Building on this foundation, Aggrescan4D (A4D), released in 2024, introduced pH-dependent scoring and refined mutational design algorithms to replicate physiological conditions better and recommend conservative solubilizing substitutions (Fig. 1) (Bárcenas et al. 2024; Zalewski et al. 2024). A4D also incorporated direct retrieval of structural models from the AlphaFold Database, enabling structure-aware aggregation analysis at the proteomic scale, even for proteins lacking experimental structures.

In this mini-review, we provide a comprehensive perspective on the Aggrescan platform, encompassing AGGRESCAN, Aggrescan3D, and Aggrescan4D, and its evolution over the past two decades. We discuss how successive iterations have addressed emerging challenges in aggregation prediction, summarize their diverse applications, and highlight current challenges and future directions that could further improve predictive accuracy, integrability, and usability.

## The Aggrescan algorithms

### AGGRESCAN – 2007

Before AGGRESCAN’s release in 2007 (Conchillo-Solé et al. 2007), existing tools, albeit algorithmically rigorous and extensively benchmarked, were based on physicochemical scales and statistical mechanics and did not incorporate biological measurements of aggregation (Fernandez-Escamilla et al. 2004; Tartaglia et al. 2005). AGGRESCAN, by contrast, uses an aggregation propensity scale based on *in cell* aggregation of mutational protein variants, rather than relying exclusively on physicochemical intuition.

The foundational dataset for AGGRESCAN was generated using an in vivo reporter assay designed to monitor the aggregation of the human Aβ42 peptide—specifically, its central hydrophobic cluster, a region known for its strong amyloidogenic behaviour (De Groot et al. 2006). In this system, Aβ42 variants were fused upstream of Green Fluorescent Protein (GFP). Because GFP must fold properly to fluoresce, aggregation of the upstream peptide prevents GFP maturation, resulting in diminished fluorescence (Wurth et al. 2002). A mutational scan of position Phe19, which was shown to strongly affect Aβ42 fibrilization, yielded 20 peptide variants. Measuring GFP fluorescence for each construct in E. coli provided a direct readout of their in vivo aggregation propensity, enabling the derivation of a residue-specific aggregation scale. This empirical scale was shown to strongly correlate with tabulated hydrophobicity scales based on octanol partitioning and the Kyte-Doolittle method, as well as β-strand propensity (Chiti et al. 2003), two key features in protein sequences associated with aggregation tendency.

**Fig. 2:**
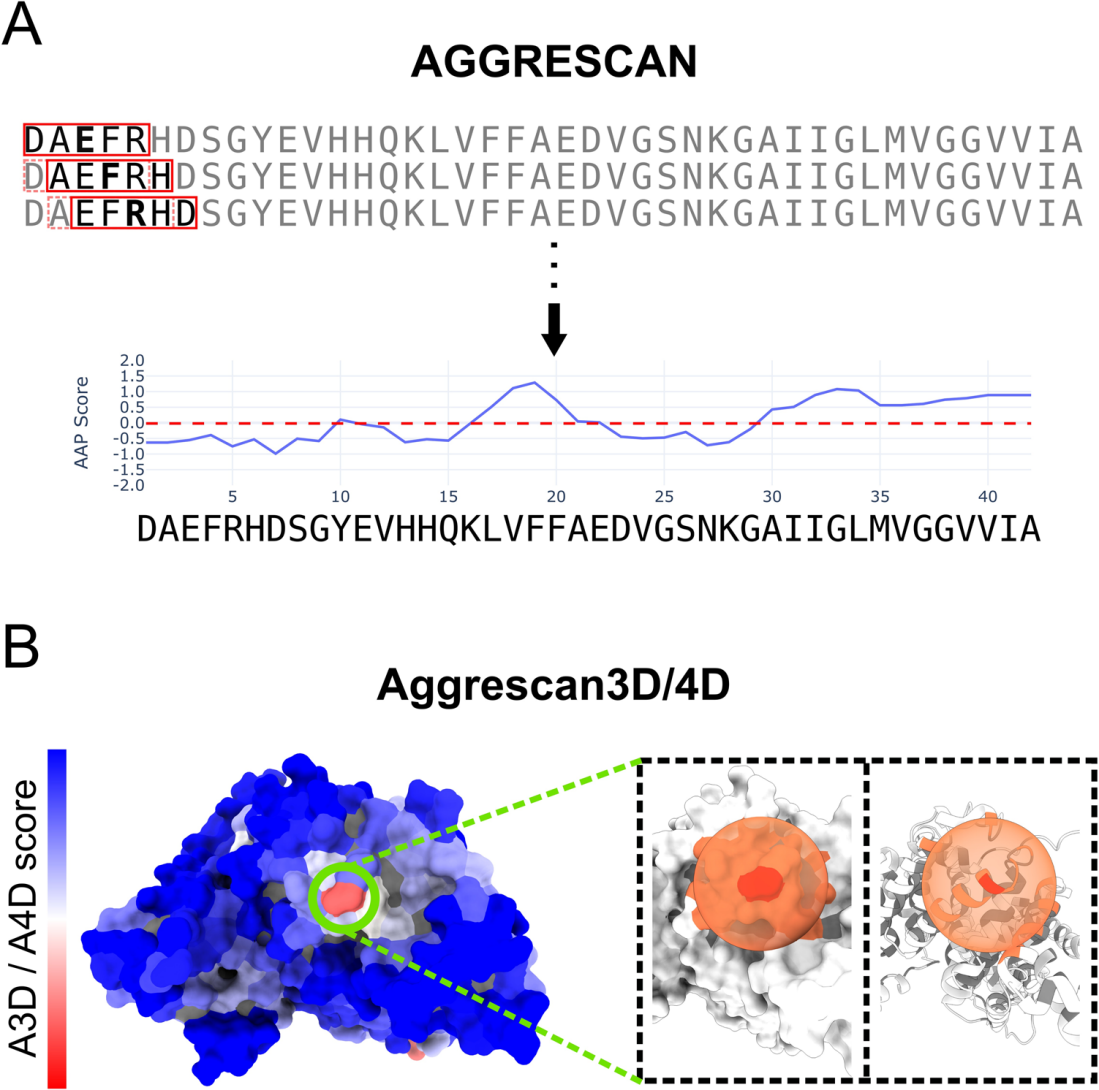
Schematic representation of AGGRESCAN and Aggrescan3D/4D aggregation calculations. (**A**) AGGRESCAN calculates a protein sequence's propensity to aggregate using an empirically derived aggregation scale and a sliding-window algorithm. The sequence of Aβ42 peptide is used as a reference. In summary, the central position of a window gets assigned the average aggregation value of all individual residues within said window. Afterwards, the window is shifted one position, and the next aggregation value is calculated. The carboxy and amino termini act as virtual residues. Residues that cannot be incorporated into a window take the value of the closest window; in this case, the value of the initial D residue takes the same value as A. Once all positions are calculated, we obtain the profile shown at the bottom of panel A, with the Average Aggregation Propensity (AAP) score assigned to each position. Regions with five or more sequential residues above the hotspot threshold (red discontinuous line) are susceptible to aggregation (in this specific case, residues 17–22 and 30–42). (**B**) Aggrescan3D and Aggrescan4D calculate the aggregation propensity of each residue exposed to the solvent based on the AGGRESCAN scale and the proximity to other solvent-exposed residues. A3D and A4D use a color code to signify the aggregation propensity of residues, with bluer shades indicating soluble positions and redder shades indicating aggregation propensity. In the example shown, the aggregation propensity of Bovine Serum Albumin (BSA) is evaluated, with residue 228 (Valine) in red (i.e., with high aggregation propensity). By default, all solvent-exposed residues within a 10 Å sphere from Val228 are considered for aggregation propensity calculations. On the right side of panel B, white residues are residues exposed to the solvent but not within the 10 Å volume for calculations, gray residues are not exposed to the solvent, and orange residues are considered for the calculation of residue 228, in red. This process is repeated for all solvent-exposed positions

The AGGRESCAN predictor incorporates this aggregation propensity scale into a sliding-window algorithm, that assigns to each residue the average aggregation tendency of the surrounding sequence context (Fig. 2A). The window size was adjusted using a known database of amyloidogenic proteins, dynamically adapting it to protein length (5 for ≤ 75 residues, 7 for ≤ 175 residues, 9 for ≤ 300 residues, and 11 for > 300 residues, respectively). To emulate the chemical environment of protein termini, the algorithm also introduces a virtual basic residue at the N-terminus and a virtual acidic residue at the C-terminus, approximating the presence of NH₃⁺ and COO⁻ groups. Finally, a value corresponding to the average of the aggregation-propensity scores, weighted by their frequency in the SwissProt database, enabled defining a hotspot threshold.

AGGRESCAN provides detailed information on a protein’s intrinsic aggregation tendency, offering residue-level annotations that map local variations in aggregation propensity across the sequence. Its primary outputs include the full aggregation profile, the identification of aggregation hotspot regions—defined as stretches of five or more consecutive residues with scores above the hotspot threshold and lacking Proline, a strong β-breaker—and an assessment of each hotspot’s relative potency. Together with the overall aggregation score of the polypeptide, these features deliver an interpretable snapshot of aggregation determinants. Beyond descriptive analysis, AGGRESCAN might also guide, to a certain extent, protein engineering by pinpointing vulnerable regions and enabling direct comparison of sequence variants. Its widespread adoption stems from this interpretability, combined with free accessibility (http://bioinf.uab.es/aggrescan/), ease of use, and computational efficiency, which together make AGGRESCAN suitable not only for individual protein studies but also for large-scale proteomic analyses.

### Aggrescan3D & Aggrescan3D 2.0–2015 & 2019

Protein aggregation predictions relying solely on protein sequence can lead to incorrect assessments of a protein's aggregation propensity, especially for globular proteins. In native conditions, globular proteins consist of a tightly packed hydrophobic core, shielded from solvent, and a predominantly hydrophilic exterior. Because the hydrophobic core is not solvent-exposed under physiological conditions, including its contribution, leads to an overestimation of the aggregation propensity of the native protein. Additionally, sequence proximity does not necessarily correspond to spatial proximity: residues that are adjacent in the sequence may lie far apart in the folded structure, while distant residues can cluster in three-dimensional space. These issues, common to early predictors, motivated the development of Aggrescan3D (A3D), a structure-informed algorithm capable of accurately estimating the aggregation propensity of globular proteins in their native conformations (Kuriata et al. 2019b, 2019a; Zambrano et al. 2015).

Aggrescan3D integrates AGGRESCAN’s empirical scale with the three-dimensional coordinates of a protein structure to identify which residues are solvent-exposed and quantify their contribution to the aggregation potential of the surface. By extending beyond sequence-level analysis, the method evolved from linear APR detection to the identification of structural aggregation-prone regions (STAPs), which correspond to surface-exposed patches that promote aggregation, independent of the residues’ sequential distance.

Conceptually, the algorithm projects the AGGRESCAN scale onto the three-dimensional structure, modulating each residue’s contribution to aggregation propensity according to its structural context. The extent of solvent exposure determines whether and how strongly a residue contributes to aggregation: fully accessible residues contribute maximally, partially exposed residues contribute proportionally, and residues with < 10% exposure are excluded from the calculation. A spherical neighbourhood is then defined around each residue’s α-carbon, and the final local aggregation score is computed from the average propensity of all residues located within this sphere, weighted by their distance from the central residue (Fig. 2B). Beyond monomeric globular proteins, the incorporation of structural information also enabled the analysis of protein complexes composed of multiple chains.

To incorporate the inherent dynamism of protein structures, Aggrescan3D integrates conformational sampling through coarse-grained molecular dynamics simulations using CABS-flex (Wróblewski et al. 2025), generating 12 conformers from the input structure and identifying transiently exposed STAPs that would remain hidden in a static crystal structure. This dynamic component improves the prediction of aggregation behaviour under close to physiological conditions.

In addition to its predictive capabilities, Aggrescan3D has become a widely used tool for rational engineering of protein solubility. Its user-friendly mutational design protocol allows researchers to introduce targeted substitutions at selected residues or to apply an automated mutation scheme that prioritizes the most aggregation-prone sites and proposes charge-based solubilizing mutations. To assess the energetic effect of these mutations, the pipeline integrates FoldX calculations (Delgado et al. 2025) providing ΔΔG estimates that help predict whether a proposed mutation is likely to destabilize the protein fold.

### Aggrescan4D – 2024

Environmental factors strongly influence protein solubility and aggregation. Building on Zamora et al.’s work (Zamora et al. 2019), which quantified how pH modulates the intrinsic lipophilicity of amino acids, we demonstrated in 2020 that these physicochemical changes could be translated into predictions of pH-dependent aggregation propensity (Santos et al. 2020). This led to the development of SolupHred, the first disordered protein aggregation predictor that incorporated pH into its scoring function (Pintado et al. 2021). Afterwards, we sought to merge SolupHred with the structural logic of Aggrescan3D. This led to the latest addition to the Aggrescan suite: Aggrescan4D (Bárcenas et al. 2024).

The incorporation of environmental pH effects enhanced the physiological relevance of the predictions, allowing for the study of protein aggregation across various contexts, including comparisons of subcellular and tissue localization, as well as health and disease states. Moreover, in industrial settings, it offered the opportunity to control aggregation simply by tuning buffer conditions rather than altering the protein sequence, thereby reducing the risk of destabilizing the fold and avoiding complications related to manufacturability or intellectual property.

Moreover, the growing availability of high-accuracy structural models, such as those produced by AlphaFold (Jumper et al. 2021; Senior et al. 2020), greatly expanded the structural landscape available for analysis. To take advantage of this resource, Aggrescan4D connects to the AlphaFold database (Varadi et al. 2022), allowing users to seamlessly access precomputed AlphaFold structures by providing the protein’s UniProt identifier.

Aggrescan4D also incorporates a new mutation protocol based on evolutionary substitution matrices. This new protocol introduces evolutionarily conserved solubilizing mutations to minimize the effects of mutations on protein structure and function. Combined with improvements to the structural engine inherited from Aggrescan3D, these developments solidified Aggrescan4D as one of the leading software tools for engineering protein aggregation tendencies (https://biocomp.chem.uw.edu.pl/a4d/).

### Benchmark and validation

The algorithm suite has been extensively benchmarked and validated. The original AGGRESCAN demonstrated that proteins predicted to have high aggregation propensities formed inclusion bodies when expressed in *E. coli*, whereas those with low scores remained soluble. This relationship extended to disease-associated proteins, including α-synuclein, Tau, the prion protein, and islet amyloid polypeptide, where predicted APRs coincided with experimentally confirmed segments critical for protein aggregation (Conchillo-Solé et al. 2007). AGGRESCAN has been extensively evaluated against other sequence-based tools for protein aggregation prediction, showing comparable or superior performance (Belli et al. 2011; Errico et al. 2025).

Aggrescan3D has also been compared with alternative aggregation predictors, demonstrating the superiority of considering the protein structure and correctly ignoring the contribution of APRs buried within the hydrophobic core of folded polypeptides. Beyond this anecdotal evidence, systematic benchmarking demonstrated significantly improved sensitivity, specificity, and precision compared to other predictors (Zambrano et al. 2015). It has also shown the usefulness of sampling protein flexibility to predict the aggregation properties of both monomeric and multimeric proteins. In 80% of the cases surveyed, the average of all conformations was higher than that of the static structure, suggesting that the starting structure may lie within a local structural minimum that underestimates aggregation risks of the ensemble (Kuriata et al. 2019b). Aggrescan3D was used by our lab to engineer, to our knowledge, the most soluble GFP variant reported to date (Gil-Garcia et al. 2018).

Finally, Aggrescan4D was benchmarked on 18 cases that met the criteria for quantitative evaluation, selected from over 300 peer-reviewed studies reporting pH-dependent changes in protein aggregation or solubility. The curated dataset and corresponding results are available in the Supplementary Material of the Aggrescan4D publication (Bárcenas et al. 2024). In this benchmark, Aggrescan4D achieved perfect predictive performance and outperformed the only other method that accounts for pH effects within a structural framework, CamSol 3.0 (Oeller et al. 2023).

## Applications

**Fig. 3:**
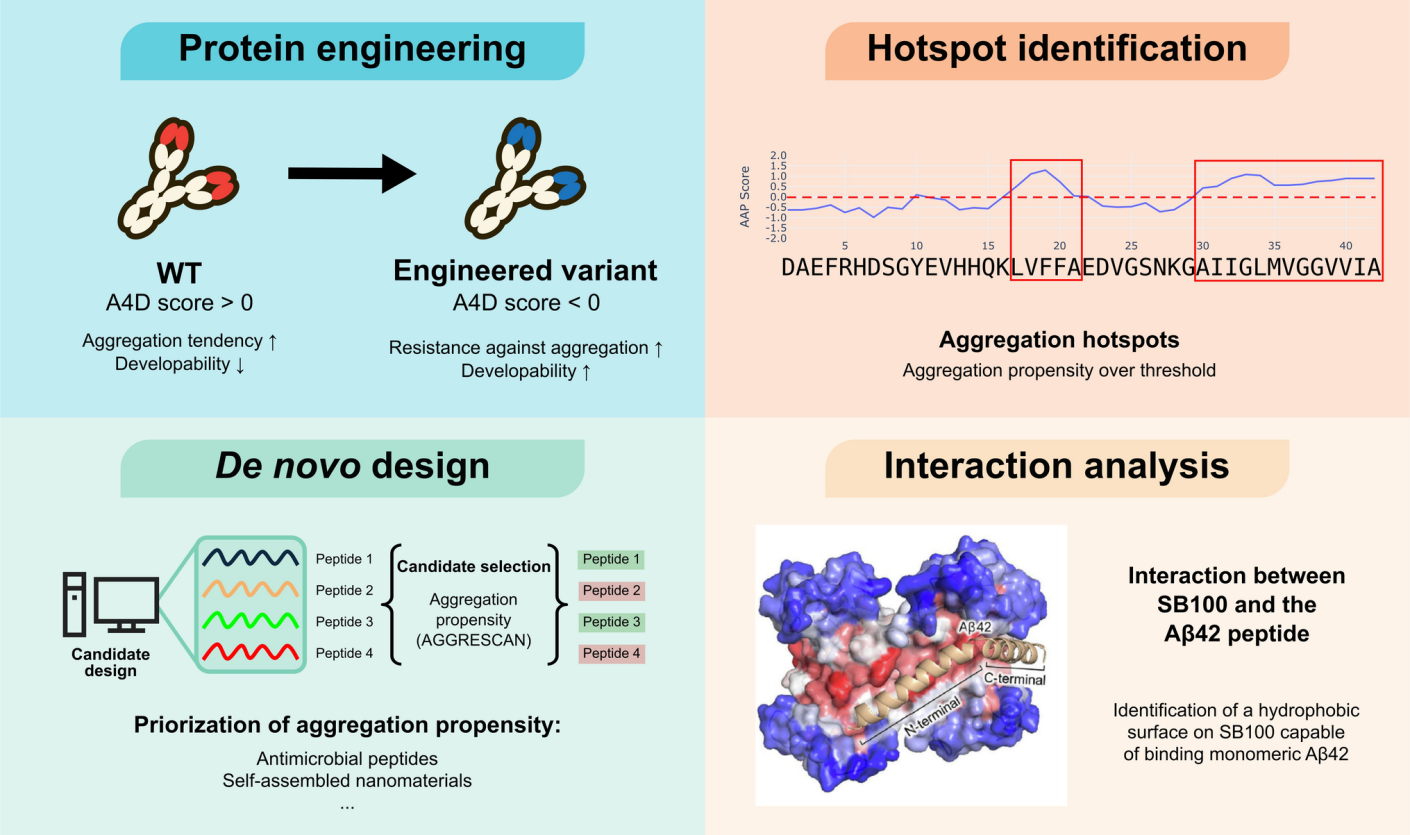
Application showcases of the Aggrescan algorithm. Aggrescan has been used to reduce the susceptibility of industrially relevant proteins to aggregate (including monoclonal antibodies). Aggrescan has also been used to identify disease-related aggregation hotspots and design peptides based on their aggregation propensity, which is closely related to the activity of antimicrobial peptides (AMPs) and self-assembled nanomaterials. Finally, Aggrescan has been used to study aggregation-related interactions by detecting hydrophobic grooves that bind to disease-related proteins and peptides, such as the Amyloid Beta 42 peptide (structure image extracted from (Figueira et al. 2022))

AGGRESCAN and its subsequent versions are powerful tools for identifying APRs. This feature has broad applicability across different disciplines (Fig. 3). Mapping APRs provides insights into the molecular determinants of aggregation, both in pathogenic settings and in physiological processes where controlled assembly is required. In applied contexts, these predictions may guide the optimization of protein solubility, stability, and developability.

One of the primary challenges in antibody development is their inherent tendency to aggregate, which significantly impacts the efficacy and safety of therapy. Aggregation undermines solubility and stability, reducing expression yields and complicating formulation and storage. More importantly, high-molecular-weight aggregates can elicit unwanted immune responses, posing severe clinical risks. Thus, evaluating the aggregation propensity of specific antibody regions offers an indirect but highly informative readout of their solubility and developability. This information can, in fact, be complemented with data from other predictive tools, such as CamSol, which estimates residue-level solubility and which, in combination with Aggrescan, yields a more complete picture of protein behavior (Ejaz et al. 2023; Fang et al. 2025; Heravi et al. 2025; Uto et al. 2025). Such multi-parametric strategies are increasingly being incorporated into de novo antibody design pipelines, helping to identify candidates with inherently low aggregation propensities before experimental testing (Fig. 3) (Ejaz et al. 2023; Yan et al. 2017). Several studies have validated these *in silico* models by expressing them in *E. coli* and conducting accelerated stability tests, confirming the promising potential of these workflows for antibody design (Fang et al. 2025; Heravi et al. 2025).

Another key application of the Aggrescan suite is the identification and characterization of APRs involved in pathogenic and physiological processes. As previously described, both environmental perturbations and intrinsic molecular factors can trigger protein misfolding and aggregation. These aberrant assemblies often impair cellular homeostasis and may ultimately lead to toxicity, as is well documented in numerous neurodegenerative diseases (Ahmad et al. 2025; Berdyński et al. 2022; Nam et al. 2025; Scialò et al. 2025). By pinpointing the sequence and structural determinants responsible for aggregation, Aggrescan tools help elucidate the mechanisms underlying aggregate-associated dysfunction and toxicity (Ahmad et al. 2025; Berdyński et al. 2022; Kaushik et al. 2021; Majumder et al. 2024; Martínez-Rubio et al. 2022). In this way, Kaushik et al. (2021), investigated cofilin-1, a central cytoskeletal regulator implicated in ALS, Alzheimer’s and Parkinson’s disease. Among four aggregation predictors tested (AGGRESCAN, TANGO, FoldAmyloid, and AmylPred2), AGGRESCAN uniquely identified nine aggregation-prone regions, demonstrating superior sensitivity. *In vitro* assays confirmed that cofilin-1 indeed forms amyloid-like fibrils. The authors then utilized Aggrescan3D 2.0, along with complementary simulations, to model the impact of cysteine oxidation on the cofilin-1 structure. These analyses uncovered a novel mechanism governing cofilin-1 aggregation.

Aggrescan can also be integrated with molecular dynamics (MD) simulations to gain deeper insight into how conformational fluctuations influence aggregation propensity. In a 2020 study, Aguayo-Ortiz R et al. (2020) examined the oligomerization of γD-crystallins (HγDC) in the eye lens, which is linked to the development of cataracts (Aguayo-Ortiz and Dominguez 2020). Evidence suggests that a mutation at W42 of HγDC (in the N-terminal domain) favors the population of partially unfolded conformers that show an increased tendency to aggregate. To investigate this, different protein variants were simulated using MD, and their aggregation tendencies were evaluated. Unexpectedly, polar and/or small residue mutations produced, on average, the highest A3D score values over the course of the simulation. The authors hypothesized that the smaller-sized side chains lead to the opening of a small pore, allowing water molecules to enter the hydrophobic core of the N-terminal, resulting in unfolding and exposure of the hydrophobic core. Coupling studies of protein dynamics with *in silico* assessment of aggregation tendency appears as a convenient way to deepen our understanding of protein misfolding diseases.

Beyond pathology, Aggrescan can also be applied to identified APRs involved in physiological functions, as exemplified by the alarmin SB100, an astrocytic protein overexpressed in Alzheimer's disease. In 2022, Figueira et al*.* demonstrated that SB100 can inhibit Aβ42 aggregation via one of its APRs, acting as a chaperone-like protein (Fig. 3). Using Aggrescan3D 2.0, they revealed a hydrophobic surface on SB100 capable of binding monomeric Aβ42, thereby interfering with the primary nucleation step and functioning as an endogenous anti-aggregation agent (Figueira et al. 2022).

Insulin is known to form fibrils under certain conditions (Surin et al. 2020), which reduces its therapeutic efficacy and can lead to tissue necrosis at injection sites (Iwaya et al. 2019)—a serious complication for patients with diabetes. Because the core amyloidogenic determinants of insulin were not fully understood and no standardized methodology exists to study its aggregation in vivo, computational tools offered a valuable starting point. In a 2020 study, Surin et al*.* applied AGGRESCAN together with several orthogonal predictors to identify APRs in insulin and insulin analogues (Surin et al. 2020). Their analysis revealed one APR in the A chain and three in the B chain. Subsequent experimental validation using HPLC–MS confirmed two of the four predicted regions. These findings provided a means for the rational design of future insulin analogues with reduced aggregation propensity, potentially improving both formulation stability and patient safety (Fig. 3).

Aggrescan predictors have also been used as *in silico* screening tools for peptides and proteins in recent biotechnology applications. In a 2025 study, Chen et al. studied aggregation-prone antimicrobial peptides (AMPs) derived from *Octopus bimaculoides*, which exert antimicrobial activity by destabilizing the bacterial cell wall (Chen et al. 2025). They used Aggrescan3D 2.0 to rank the aggregation propensity of 96 peptides and identified Oct-P2 as the peptide with the highest A3D score. Both *in vitro* and cellular assays validated that this peptide aggregates in the presence of bacterial DNA, resulting in reduced viability of Gram-negative bacteria. Mechanistically, the study revealed a novel mechanism by which aggregation-prone antimicrobial peptides, such as Oct-P2, can effectively kill bacteria by aggregating with bacterial DNA to disrupt transcription and translation, offering a promising new strategy for developing antimicrobial agents against resistant pathogens. This work also demonstrates Aggrescan’s value in computational triage to prioritize peptide sequences with desirable self-assembly profiles.

Aggrescan is increasingly used in the de novo design of new proteins or peptides (Minich et al. 2022; Seyedhosseini Ghaheh et al. 2022). Two recent works used AGGRESCAN and Aggrescan3D 2.0 to guide the site-directed mutagenesis of the alcohol dehydrogenase from *Rhodococcus ruber* and of Reteplase, a recombinant thrombolytic enzyme, to improve their solubility and stability. The authors identified critical APRs and rationally designed soluble variants of the proteins. These redesigned proteins displayed increased expression yields in *E. coli*, improved solubility, and enhanced enzymatic performance relative to their wild-type counterparts. These results demonstrate how controlling aggregation improves folding efficiency and in-cell stability, ultimately increasing biomanufacturing output and strengthening the overall developability of biologics—two key requirements in pharmaceutical protein production.

Finally, Aggrescan has proven valuable in the field of self-assembling biomaterials (Sunil et al. 2025). Many next-generation biomaterials leverage the innate ability of peptides to form cross-β architectures, resulting in mechanically strong, water-retaining matrices. In a 2025 study, an ovoalbumin-derived peptide was shown to self-assemble into a hydrogel with antioxidant activity *in vivo*. Computational analyses—including AGGRESCAN—predicted a high tendency for aggregation and amyloid formation, consistent with the peptide’s ability to form ordered fibrils. This demonstrates how amyloid-like aggregation, often linked with disease, can be harnessed to create stable, functional, and biocompatible biomaterials (Fig. 3). Looking ahead, integrating AGGRESCAN into biomaterial development pipelines could accelerate the rational design of functional assemblies by enabling the predictive tuning of their aggregation behavior.

## Discussion

As our understanding of protein aggregation deepens, the tools we use to investigate and manipulate this process must evolve accordingly. Since its debut in 2007, AGGRESCAN has progressed from a pioneering sequence-based predictor into a versatile platform that incorporates structural context, conformational dynamics, and environmental conditions (Fig. 1). This evolution has enabled its application across diverse areas—from biopharmaceutical formulation and industrial enzyme engineering to mechanistic studies of pathological aggregation and the rational design of de novo proteins and peptide-based biomaterials (Fig. 3). Globally recognized and cited over 1,500 times, the Aggrescan suite has established itself as a valuable and reliable resource for researchers worldwide.

Despite the substantial advances achieved with AGGRESCAN, several limitations remain that warrant further development of the platform. Current stability calculations and mutation impact predictions rely on simplified energetic and structural assumptions that, while computationally efficient, may not fully capture the complex interplay between folding, conformational dynamics, and aggregation, underscoring the need for refinement supported by systematic experimental validation. Moreover, the availability of reliable, well-annotated experimental datasets—particularly those reporting pH-dependent aggregation or solubility behavior across defined structural states—remains limited, constraining rigorous benchmarking, comparative assessment, and effective model optimization. This scarcity also hampers the incorporation of additional biochemical variables, such as post-translational modifications or context-dependent environmental effects. Addressing these limitations will be essential to further improve the accuracy, scope, and impact of the Aggrescan suite.

One exciting frontier lies in integrating machine learning and artificial intelligence. While Aggrescan is grounded in empirically derived biophysical principles, the rise of large-scale datasets of protein aggregation data—including deep mutational scanning results and proteomic aggregation profiles—provides an opportunity to complement rule-based scoring with data-driven prediction. This is particularly relevant in complex edge cases, such as regions that transition between ordered and disordered states or multi-domain proteins, where context-specific aggregation is poorly captured by static models. In this scenario, closer integration with experimental high-throughput data would benefit predictor refinement, either as validation benchmarks or as machine-learning training data. This approach would also facilitate the establishment of feedback loops between computational design and empirical testing.

Another area of development is the modeling of post-translational modifications (PTMs), such as phosphorylation, ubiquitination, glycosylation, and acetylation. These modifications can profoundly reshape protein solubility, electrostatics, conformational dynamics, and ultimately aggregation behavior. While Aggrescan4D allows users to simulate structural exposure and charge-state changes, future versions could include explicit modeling of PTMs at the sequence and structural levels. This would be especially relevant in pathological contexts such as neurodegenerative diseases, where aberrant phosphorylation of tau or α-synuclein occurs, as well as in industrial applications, where glycosylation determines the manufacturability of therapeutic proteins.

Enhancing Aggrescan’s treatment of protein dynamics is another key objective. Current flexibility modeling relies on CABS-flex simulations. However, integration with full-atom molecular dynamics simulations could provide more granular insight into transient aggregation-prone states, domain movements, and long-range effects of mutations. Such coupling would also allow aggregation propensity to be evaluated under specific physicochemical regimes, including stress conditions such as elevated temperature, oxidative environments, or macromolecular crowding.

A more ambitious, yet increasingly relevant, direction is to extend Aggrescan beyond isolated proteins to contexts in which aggregation propensities govern both soluble-to-insoluble phase transitions (classical protein aggregation) and soluble-to-soluble phase transitions, such as liquid–liquid phase separation (LLPS). While Aggrescan is not intended to predict condensate formation per se, its ability to identify sequence- and structure-encoded aggregation-prone regions could provide mechanistic insight into how condensates mature, age, or transition toward irreversible assemblies. In particular, aggregation-prone motifs that are silent under dilute conditions may become locally enriched or transiently exposed within condensates, acting as nucleation points for gelation or solidification. In this framework, Aggrescan would complement existing LLPS models by helping to delineate the molecular boundary between reversible condensation and pathological aggregation.

From an implementation perspective, improving accessibility and scalability is essential. Although web servers are heavily utilized, large-scale proteomic studies, industrial high-throughput design pipelines, and computational protein engineering labs require faster, parallelizable, and easily deployable solutions. Standalone versions of AGGRESCAN and Aggrescan4D are currently under development, with plans to use them in distributed computing networks and academic supercomputers, helping broaden their adoption and support real-time design in large teams or across institutions. Aggrescan can also be integrated into generative AI tools, such as sequence-generation algorithms, to avoid APRs or enforce folding determinants, thereby supporting de novo protein engineering. Finally, integrating Aggrescan into community-driven databases and public aggregation atlases could create shared repositories of structure–function–aggregation relationships.

In summary, as protein aggregation continues to pose challenges in medicine, biotechnology, and basic biology, tools capable of accurately predicting, interpreting, and manipulating this process will become increasingly important. With a strong foundation in experimental validation, structural integration, and user accessibility, the Aggrescan suite is well-positioned to meet the evolving needs of protein science and help shape the next decade of protein aggregation research.

## Data Availability

No datasets were generated or analysed during the current study.
